# Our Summer at Maplewood—Chapter VIII.—Mrs. Rose’s Omelet

**Published:** 1875-09

**Authors:** 


### CPAPTER VIII.

### MRS. ROSE'S OMELET.

"Broiled chicken for breakfast—how nice!" said Cousin Emmeline, as she lifted the silver cover from the dish before me. "What a pity so few cooks can broil well! I've had more difficulty in the broiling department than in any other."

"Broiling, Emmeline, when properly done, is a very satisfying method of cooking steaks, chops, chicken, etc.—satisfying to the eater thereof, I mean. But the poor housewife who broils herself, as well as her meats, in the laborious effort to attain perfection, does not perhaps enjoy it to the same extent. With a suitable fire and other conveniences, broiling is not at all difficult. But with the average range or cooking-stove it is no easy matter, as the fire is seldom in right condition at the important moment. And my recommendation to women who toil and broil and suffer, to bring the broiled steak or chop to the table in perfection, is to try this method. At the time of placing the steak over the fire, put into the oven a dripping pan large enough to hold the steak without folding. As soon as the steak is lightly browned on one side, turn and brown it on the other; then transfer instantly to the hot pan and oven, where, if it be thick it will need to remain from five to ten minutes, according to the state in which it is to be served. Serve on a heated platter, and season after removing from the oven. This method relieves the cook, saves all the juices of the meat, and prevents it from burning on the outside while it remains raw within. No one can tell a steak so cooked, from one finished on the gridiron; and those who have tried both methods, find the hot-oven finish far superior. But it will not answer to have the oven warm, merely. It must be hot. Or, steak, chops, etc., may be broiled—literally broiled, in this way: Set your spider on the stove and let it get smoking hot. Put in no butter, nor any kind of grease. Have your meat previously prepared by trimming off all pieces of bone, gland, superfluous suet, and tissue that will bind the edge and make it turn up. Lay it carefully and smoothly in the spider. It will stick fast at first, but as soon as it is browned, it can be loosened with a knife. When juice begins to appear turn it over, and let the other side brown the same as the first. Press closely to the pan when turned, and turn as often as is necessary to save the juices and cook the steak properly. Lamb chops should always be broiled on a hot pan, unless they are very much trimmed, that is, unless the small piece of lean tender-loin is separated entirely from the bone and fat, which should be rejected. These trimmed chops may be broiled over a clear fire, in a wire gridiron, such as is used for oysters. And when so cooked are very delicious. The rejected bone and fat can be served in a stew, or in the soup kettle. But it would be no more wasteful to throw them away, than to retain and broil altogether, as is usually done. For in the latter case the result is generally a badly damaged chop, smoked and burned from the dripping of grease—an unsightly, awkward piece, from which nine persons out of ten select and eat the small bit only, leaving the rest upon their plates to be thrown out with the scraps from the table. But in my cook-book, in connection with this recipe for broiling, I intend to put in staring capitals; The broiling pan must be kept piping hot all the time the meat is cooking."

"Your labor-saving expedients," remarked Emmeline, may do well enough with chops and steaks, and may probably prevent them from being burned or underdone. But by no substitute for the gridiron, or by no make-believe method of the sort you recommend, could a broiled chicken be produced, that would begin to approach this in excellence."

"Ah, ha!" said Alice, gaily, "you are in error there. This chicken has never touched, nor seen live coals. It was done entirely in a hot oven, after one of Cousin Kate's labor-saving methods. This is the way it was cooked: The chicken was properly prepared for broiling, by being opened down the back, washed in cold water, and wiped dry with a soft cloth. The breast bone was then flattened with a mallet, the wings were twisted back to leave the breast exposed, and the chicken was placed, skin up, in a dripping pan, and pressed close to the pan to make it lie as flat as possible. After being thus fixed, I put it into a hot oven and shut the door. In about five minutes I heard a sputtering inside, and began to think something was wrong; but Cousin Kate dissipated my fears by assuring me it was cooking nicely. From time to time I peeped into the oven, just to see that it was not burning, and at the expiration of twenty or twenty-five minutes, I placed the chicken on a heated platter, seasoned it with pepper, salt and butter, and here it is before us."

"Incomparable as a broiled chicken!" was Emmeline's response.

"So thinks my fastidious mama? But would you have thought so had you known before you tasted it, that it was oven-cooked?"

"I do not think my prejudice in favor of broiling could prevent my appreciating so perfectly cooked a chicken as you have served us this morning, Alice. But why do you not season, and baste it with butter, before you put it in the oven? I would do so; and would also put water in the pan, to keep it from burning."

"There you would make a great mistake," said Alice, decidedly. "The pan must be dry, so that the chicken will brown on the bottom; and the chicken must be dry, so it may broil, instead of steam and stew. Besides, if the chicken was basted with butter it would brown with less heat; and Cousin Kate says the secret of success in this method, is in having the oven just as hot as the chicken will bear without burning. It would by no means be the same, in appearance or taste, if done in a slow, or even a moderate oven."

"All birds that are good broiled, are better when cooked in a hot oven in the manner Alice has just described," I remarked, when the appearance of Tom with the mail interrupted the conversation. Among my letters was one from my friend Mrs. Rose, containing some hints for my cook book. In regard to quail, she wrote:—

"I think there is no bird more delicate than a quail broiled in this manner: Lay the bird on a gridiron, and, when it begins to brown, dip it into butter, seasoned with salt and pepper. Continue to broil and dip, until it is done brown—a nice yellow brown all over. Serve hot."

"You don't approve of that, Kate, do you?" interrupted Emmeline, it seems to clash with some of your theories."

"At all events, Emmeline, I shall not condemn without trying it. And perhaps the use of the butter here to hasten the browning, may be a good thing, as otherwise the bird might be too much done before it was properly browned. But to return to the letter.

—"Wild ducks should be cooked as soon as possible after they are shot. I know that large quantities are sent to gentlemen in London, by their friends in this country. But by the time they reach them, I doubt not, the English gentry think very much as Mrs. M. did, especially if their ducks are cooked in the same manner her's were. Her husband's brother sent her from Washington, a pair of canvas-backs for which he paid $10; but she didn't think them as good as a pair of tame ducks she could buy in her own town for ten shillings. I asked her how she cooked them. She said she stuffed them with bread dressing, seasoned with onion, and baked them an hour and a half. Now, this is my recipe: Draw out the entrails and rinse the ducks; but don't soak them, as some ignorant cooks do, or you will lose the juices. Rub inside with salt and pepper, and put in each duck a piece of butter the size of an egg, and a teaspoonful of red wine. Roast twenty or twenty-five minutes. By no means allow the birds to be moved while roasting, lest their juices be spilled. When done they will be full of a bright, red gravy. Remove carefully, too, and serve on a hot dish. I presume Mrs. Rose's recipe is an excellent one, as she lived several years on the Eastern shore of Maryland, where they are famous for serving up such epicurean luxuries as canvas-back and terrapin, in the most approved manner. But let us get on with the letter: 'I hope you will devote a chapter in your book to omelets. Did I ever tell you how it took me seven years to learn how to make an omelet? Now, I don't mean you to understand that I devoted my undivided time and attention to the study of omelets alone, during that period. But as occasion offered, during seven years I tried various and varying recipes from at least a score of cook books. As directed, I beat the eggs together at one time, and beat them separately at another time. I put cream in some, and in some I minced ham, parsley or onion. I made omelets as light as a puff and as dry as a husk. I produced omelets soft and frothy, as well as omelets flabby and leathery. I concocted omelets that were certainly first cousin to scrambled eggs; and omelets more nearly related still to baked custard. But the omelet I was striving for—the omelet I had found in my girlhood days at first-class hotels and tables where I could not take the cook aside and ask how it was made,—this ideal omelet was not to be evolved from all the materials prescribed by these cook books. Discouraged and disappointed I rested from my labors; and, Harry and I ate eggs boiled, poached and scrambled. But at last I obtained the object of my endeavors. One morning I found upon the table of an intimate friend *my omelet*. 'Did you make this?' I asked, eagerly. 'Tell me exactly how you did it. Begin at the beginning, and let me know every twist and turn of the process.' 'Why,' she answered, with a complaisant smile, 'it's the easiest thing in the world. For my family, I take five eggs. I break them into a bowl, and beat them with a spoon, lightly, until I can dip up a spoonful. I beat them only enough to break them up, and render them manageable. I have ready a little minced parsley, salt and pepper. I place my omelet pan, which is never used for any other purpose, with an ounce of sweet butter in it, on the stove; and as soon as the butter is hot,—be careful not to let it brown,—I pour in the omelet, rinse the egg from the bowl with three teaspoonfuls of sweet milk—cream is better—and pour it over the omelet in the pan. I then sprinkle in salt, pepper, and the minced parsley, and set it to cook where it will have moderate heat. While cooking I stir it gently with a fork, and when almost done, place it for a moment where the heat is a little quicker, so it may brown lightly on the bottom. When ready to serve, I slip a knife blade under one side, holding the pan slightly tipped, and fold the omelet over, leaving half the pan naked, and the omelet in the shape of a turn-over pie. And now comes the only difficult part of the operation—that of turning the omelet from the pan upon the platter. Don't forget to heat the platter,—a cold omelet isn't fit to eat. Hold the pan close to, and partly over it. Give it a sudden dip—the pan, I mean—and a gentle flop, and there lies your omelet—just where you desired—a neatly shaped, plump little thing, about three inches thick, moist, light, and, in my judgment, the best omelet that can be made.'

I went home happy, intending to surprise Harry next morning with my ideal omelet—the dainty dish he had heard me talk about and sigh for so often. But, alas, for human expectations! I made the omelet, and hesitated not until the time to stir it came. Then a score of perplexing questions arose. 'Stir gently two or three times,' was what my friend had said. But what *is* stirring gently? Does to stir gently mean to stir slowly? And how soon should I begin to stir? And why stir with a fork? As no one was present to answer these and the other questions that suggested themselves, I seized a fork and began stirring slowly, round and round. But the tines of the fork scratched over the bottom of the spider, and that seemed the only result of my labor. After waiting awhile, I stirred again. This time I found the egg adhering to the pan, and stirred more vigorously, thinking by so doing to accomplish the desired object. But when I attempted to turn the omelet, it stuck fast, and wouldn't fold; so I worried over and scraped at it, until all form and comeliness were lost, and it was nothing but a shabby specimen of scrambled eggs. Then I generously left it all for Bridget's breakfast.

In despair I went to my friend, who was surprised at my failure; but, to my great delight, she said, 'stay to lunch, and I will make an omelet so that you may see just how it is done.' I watched carefully every movement, and kept saying, '*I* did precisely so,' until stirring time came. Then my interest grew intense. Placing the omelet in the pan, she sat it over moderate heat, and waiting just about a minute, dipped in the fork. Finding the egg had set, or slightly cooked upon the bottom of the pan, she lifted or picked it up, here and there, at various points,—each time raising her fork entirely out of the omelet, and dipping it in at another point. Light dawned. I saw the secret of the whole thing was in this peculiar moving of the omelet as it cooked; and in my amazement, I exclaimed —'and *that* performance you call stirring? I might have tried till dooms-day, from your directions, and wouldn't have cooked the omelet properly? 'Well, it it isn't stirring—what is it? What would *you* call it?' 'Lifting would express the idea more correctly,' I replied. 'It is lifting the cooked egg that adheres to the pan, so that the uncooked egg may take its place. The egg forms a thin layer or cake on the pan, and dipping the fork in here and there at various points and lifting, loosens the whole mass. The effects produced by stirring and lifting are very different, and in the manufacture of an omelet are very apparent.' When a second layer had cooked, the lifting operation was repeated, and so on until the cooked egg filled the pan, and there was no more to be let underneath. In this delicate process of lifting lies the whole secret of the operation. Done in this manner, the butter is not stirred away from the bottom of the pan, and enough remains to allow the omelet to brown nicely, and prevent it from sticking. Since that memorable day I have never failed in making an omelet that gave me entire satisfaction.'

"I had no idea," interrupted Emmeline, "such mysteries were hidden away in an omelet. Few of us, I fear, would labor as Mrs. Rose did to discover them."

"No," I replied, "the most of us are too lazy or too careless to do so. We accept the indifferent food that is placed before us, and grumble at the cook for selecting such unpalatable dishes; but never dream the wretched preparations can be improved upon. Mrs. Rose is an exceptional woman who never rests satisfied with a dish that she thinks she can in any way improve. Her omelets, I know, from having seen and tasted many of them, are unsurpassable. And I am not willing to believe any woman such a natural stupid as to be capable, after reading the minute description given in these graphic experiences, of making a failure of an omelet. Her letter continues:

'What do you think of this for salad dressing? I have copied it from a recently published cook-book by a very popular writer. Here is the dressing for "two full-grown chickens and three bunches of celery:" 'Two cups boiling water, two table spoonfuls of corn-starch wet with cold water, two table-spoonfuls of oil, one cup of vinegar, two tea-spoonfuls of made mustard, one great spoonful of fat, skimmed from the liquor in which the fowls were boiled, three raw eggs, three hard boiled eggs, one tea-spoonful powdered sugar, one tea-spoonful salt, one tea-spoonful pepper and one tea-spoonful Worcestershire sauce.' I prefer my salad with a less elaborate dressing. But there is no accounting for tastes, and there are persons, perhaps, who would relish such a concoction. This is the best recipe I know for oil dressing for chicken or other salad: For three chickens take the yolks of eight fresh eggs. Put them in an earthen bowl, and with a silver spoon stir gently and slowly, round and round. Don't beat them. Drop in oil, drop by drop at first, then in small quantities, and very slowly, until in the course of an hour you have used the whole bottle of oil. At last add three table-spoonfuls of vinegar, two of made mustard, a tea-spoonful of salt, and cayenne pepper to taste, continuing the stirring slowly all the while. Prepare the chickens by boiling whole. Salt the water in which they boil, very slightly. Cut the chickens when cold into small bits, three-fourths of an inch in length, and reject the skin and fibrous pieces. Cut the celery in pieces an inch long, and have two-thirds as much celery as chicken. Put the chicken and celery, when thus prepared, in an earthen bowl, and mix well with a fork. Add the dressing just before serving.'

"I have no doubt Mrs. Rose's recipe for salad dressing is very nice, and for those who like oil, unexceptionable. In my cook-book, in connection with hers, I will give the following recipe: Take the yolks of eight fresh eggs and one gill each of strong cider-vinegar and water. Heat, to boiling, the vinegar and water, and pour slowly over the beaten yolks, continuing to beat while adding the hot liquid. Put the mixture in an earthen bowl, and set on the range or stove, in a pan of boiling water. Let the water boil around the bowl until the contents are cooked. Stir frequently, and, after it begins to thicken, continuously, until done, when remove at once, and continue the stirring for a minute, while cooling. Do not cook it enough to curdle it. Add mustard, pepper and salt, to taste. When cold, thin to the consistency desired, with sweet cream. If cream can not be had, add an ounce of sweet butter while the dressing is hot; and use sweet milk to thin it, when cold. This dressing may be kept in a cool place several days. But when so kept, the cream or milk must not be added, till wanted for use. It is excellent for cold-slaw, lettuce, etc."

"Kate, don't forget to give a recipe for frizzling beef, in your book," said Emmeline, as I laid down Mrs. Rose's letter. "Frizzled beef is a dish much used in warm weather, especially by country people, and, although so simple, it is often unfit to eat by reason of being badly prepared. Instead of being shaved, the beef is cut in thick slices or chunks, and the outer rind left on, which gives it, when cooked, a strong, rank taste. It is then boiled in a quantity of water until all the juice is extracted and the meat rendered tough and insipid, and flour and strong butter are added to make gravy; or the water is allowed to boil away, and is replaced with skimmed milk. As fond as I am of frizzled beef, I confess I have no relish for it when prepared in the ordinary slip-shod method."

"I am glad you mentioned this delicious breakfast dish. You shall have some in a few days, cooked according to my recipe. Here it is: Cut away all the rind, or dried skin, from as much of the meat as you wish to use. It can then be shaved or sliced easily, and as thin as desired, if a sharp, thin-bladed knife be used. Put a piece of sweet butter in the pan in which it is to be cooked; and when the butter is boiling hot, throw in the shaved beef. Place over a quick fire, and with a fork stir it constantly, to prevent burning. As soon as it looks frizzled or cooked, remove to a cooler place. Dash in a spoon full of flour. Mix well by stirring. Then add a little sweet milk. The amount of butter and milk used must be regulated by the quantity of meat, as in frizzled beef one wants no gravy independent of beef—the beef and gravy should be so assimilated as to render a separation almost impossible. Beef will cook or frizzle by this method in two minutes. Dried mutton, veal, etc., are very nice when cooked in this way. Many persons think a little dried liver shaved in the same manner as the beef, and cooked with it, improves the dish. Be careful to have all mould and outside skin pared or scraped off before putting the meat in the pan, as a very little mould will impart a disagreeable flavor to a large dish of frizzled beef."

"But, Cousin Kate," interrupted Alice, "Ma appears to take so much interest in culinary matters this morning that I'm afraid, unless some one makes a move, the greater part of the day will pass before we finish breakfast." And she left us and tripped lightly up stairs to her room.

After her departure, I said to Emmeline, "I have a letter here from my old friend Jennie—I mean Mrs. Douglass—in whieh she writes:—'Be not startled if we should surprise you some fine morning, by dropping in upon you at Maplewood. How charming it will be to find you in the old home to welcome us. Don't think of going sooner than you intended because of our anticipated return. Gerald joins me in insisting that you and your friends, shall remain, as you proposed, till autumn. You mention these friends simply as 'Mrs. Richmond and daughter.' Have I ever met them? The name has a familiar sound; and yet I can't recall them.'

"How annoying!" said Emmeline. "To be turned out of such comfortable quarters before we get fairly started at our literary labors, is really too bad. But, of course, we must go. I couldn't think of remaining, under all the circumstances."

Questioning in my mind what mystic meaning might be hidden in the latter part of her sentence, I replied: Don't decide upon anything hastily; and say nothing to Alice, at present, about the matter. Because, as Jennie writes, Gerald's moods may change—he is so restless and unhappy—and with them, all their plans. If they decide positively to come home, she promises to give ample notice before they sail. I dare say, however, they'll remain abroad another year."

---

INTRODUCING MEDICINES INTO THE SYSTEM BY GALVANISM.—According to Herman Munk, the failures of the various attempts to convey liquid medicaments into the living human body by means of the galvanic current, have been because the current has been sent in one direction alone; as he has found that, if a moist, porous body, between liquids of various conductivity, be traversed by the current, the speed of the conveyance of the liquid into this body rapidly diminishes, and soon becomes zero. If, under the same circumstances, the current is reversed, after a short interval, the liquid enters anew from the now positive electrode. By frequently repeating this reversal large quantities of the liquid can be introduced. Mr. Munk has in this way transferred fatal quantities of strychnine solution through the unbroken skin of young dogs, and has introduced chinin and iodide of potassium into his own arm in such quantities as to be readily detected in the excreta. The essential points, therefore, in such operations are that the liquid substance be placed at both electrodes, and that the direction of the current be frequently reversed.

